# *Gynura procumbens *Improved Fertility of Diabetic Rats: Preliminary Study of Sperm Proteomic

**DOI:** 10.1155/2018/9201539

**Published:** 2018-09-30

**Authors:** Khaidatul Akmar Kamaruzaman, Wan Mohd Aizat, Mahanem Mat Noor

**Affiliations:** ^1^Centre for Biotechnology and Functional Food, Faculty of Science and Technology, Universiti Kebangsaan Malaysia (UKM), 43600 Bangi, Selangor, Malaysia; ^2^Institute of Systems Biology (INBIOSIS), Universiti Kebangsaan Malaysia (UKM), 43600 Bangi, Selangor, Malaysia

## Abstract

*Gynura procumbens* (GP) is a medicinal herb that has long been known as anti-inflammatory and antihyperglycaemic. Recently, this herbal extract has been associated with a profertility effect, suggesting its applicability in treating both diabetes and male infertility. In this study, the effects of GP aqueous extract (GPAE) on diabetic rats were investigated through evaluating testes histology and androgen hormone levels as well as the implantation sites of female rats on copulation with the treated male rats. Three dosages of GPAE were used (150, 300, and 450 mg/kg), and there were three control groups [normal, diabetic, and metformin-treated diabetic]. Testes histology, androgen hormone levels, and number of implantation sites of the GPAE-treated groups matched those of the normal group in contrast to the diabetic and metformin-treated diabetic controls. Sperm proteomics analysis identified 666 proteins, but only 88 were consistently found in all the control and 450-mg/kg GPAE-treated groups. Four proteins, including cysteine-rich secretory protein 1, carboxylesterase 5A, zona pellucida binding protein, and phosphatidylethanolamine-binding protein 1, were significantly upregulated with GPAE treatment compared with the diabetic control, matching the protein levels of the normal group. These proteins were mainly involved in sperm maturation, sperm capacitation, and sperm-egg interaction, suggesting that GP treatment was able to restore the fertility of male diabetic rats at molecular protein level. In conclusion, GP treatment effectively treats infertility of male diabetic rats, possibly through the upregulation of proteins related to sperm maturation and sperm-egg interaction.

## 1. Introduction

Diabetes mellitus is one of the most prominent public health problems in modern societies and is rapidly increasing, specifically among those of reproductive age. According to Malaysia's National Health and Morbidity Survey [1], approximately 17.5% of individuals aged 18–36 years have diabetes mellitus, which is equivalent to 3.5 million individuals in Malaysia. Diabetes has negative effects on the male reproductive system, including testicular function, sperm maturation, and sexual hormone alteration [2]. Approximately 80% of males with diabetes mellitus experience frequent loss of libido as well as erectile and sexual dysfunctions [3]. Diabetes has also been shown to alter androgenic hormone levels, such as those of luteinising hormone (LH), follicle stimulating hormone (FSH), and testosterone [4]. High blood glucose induces a change in Leydig cells and pituitary-testicular axis, leading to decreased LH level and consequently testicular impairment [5]. Prolonged hyperglycaemia may also lead to the overproduction of ROS, which results in the disruption of spermatogenesis [6]. Hence, an effective treatment is urgently needed to overcome both hyperglycemia and fertility problem in diabetic patients.

A drug called metformin has been frequently used in treating hyperglycaemia; however, it has been found to be ineffective against infertility in diabetic patients [7]. Hence, studies are now shifting focus towards alternative herbal treatments to treat these problems. One alternative is the herb* Gynura procumbens *(GP). It is a medicinal plant of the Asteraceae family, known for its beneficial therapeutics effects, such as treating fever, rashes, hypertension, and diabetes mellitus [8]. This evergreen shrub grows extensively in Southeast Asia and is locally known in Malaysia as* Sambung Nyawa*. Several studies have reported that this herb possesses anti-inflammatory [9], antihypertensive [10], anti-cancer [11], and antihyperglycaemic [12, 13] activities and recently has been recognised as a profertility factor [14]. The administration of GP aqueous extract (GPAE) was reported to reduce blood glucose levels significantly and increased sperm quality [14]. Despite these findings, the effects of GPAE on diabetic male fertility health as well as sperm proteins have not been thoroughly elucidated.

The impact of herbal treatments on sperm proteins has been investigated in a few previous proteomics studies. For example, a two-dimensional gel electrophoresis (2DGE) study has reported that* Centella asiatica* (*pegaga*) extract caused modifications on sperm protein levels associated with energy generation process and sperm motility [15]. Additionally, administration of* Lunasia amara* Blanco (Sanrego) at 60 mg/kg also showed that the expression of sperm proteins involved in energy metabolism, sperm motility, amino acid metabolism, and cell signalling were highly increased, suggesting the herb profertility potential [16]. While most sperm protein studies have used 2DGE-based proteomics, another approach called shotgun proteomics, also can be performed whereby proteins are quantitatively compared on the basis of peptide signal intensities from mass spectrometry [17]. This approach provides higher identification of sperm proteins [18, 19] compared to 2DGE-based. However, application of shotgun proteomics in understanding the effect of herbal treatment especially GPAE on sperm proteins in diabetic patients is still limited and further experimentation is required.

In this study, the effects of three different doses of GPAE (150, 300, and 450 mg/kg per body weight) on spermatogenesis, hormonal levels, and implantation sites were investigated to further comprehend the ability of this herb to improve fertility in diabetic rats. Furthermore, shotgun proteomics were also conducted to analyse sperm protein changes due to the treatment which could highlight molecular significance related to the use of GPAE in diabetic rats.

## 2. Material and Methods

### 2.1. Preparation of GPAE

GP leaves were grown and collected from the Universiti Kebangsaan Malaysia glasshouse in Selangor, Malaysia (2.9300°N, 101.7774°E), and deposited in local herbarium with the voucher number, 40343 (KAK 01 (UKMB)). GPAE was prepared as described by Kamaruzaman and Mat Noor [20]. Briefly, 2.0 kg of GP leaves was dried in the oven at 48°C for 72 h. After drying, the leaves were ground to fine powder and mixed with water with the ratio of 1:20 before incubation in a water bath, at 60°C for 3 h. The extract was later filtered and freeze dried.

### 2.2. Diabetes Type I Induction and Experimental Design

This study was approved by the Animal Ethics Committee of Faculty of Medicine, Universiti Kebangsaan Malaysia (FST/2013/MAHANEM/31-JAN./492-FEB.-2013-FEB.-2015). A total of 42 proven fertile male 8-week-old Sprague-Dawley rats (*Rattus norvegicus*) were used in this study, and they were randomly divided into six groups. Three of the groups were considered as control groups: normal control (nontreated nondiabetic), diabetic control (nontreated diabetic), and metformin-treated diabetic control groups. Another three groups were considered as the treated diabetic groups with varying dosages of GPAE: 150, 300, and 450 mg/kg per body weight, respectively. These doses were chosen based on previous studies [13, 14] and also to determine the best dose among these three in treating both hyperglycaemia and fertility health.

Type I diabetes model was chosen for this study, using the chemotherapeutic drugs, streptozotocin (STZ). STZ is toxic to pancreatic *β*-cell, eventually induced inflammation in the pancreas causing diabetes condition in rats. STZ-induced hyperglycaemia was described as a good experimental model for type I diabetes [21]. All five groups except normal control were induced once, by a single intravenous injection of 50 mg/kg per body weight STZ at the root of the tail after overnight (12 hours) fasting. The STZ solution was prepared immediately prior to injection by dissolving the drug in a fresh, cold citrate buffer, pH 4.5. After 72 hours of induction, the fasting blood glucose level were measured using glucometer (Accucheck Performa). Rats possessing blood glucose levels of ≥ 13 mmol/L were considered diabetic. All rats were fed standard pellet diet and given access to water* ad libitum*. Subsequently, all treatments were given via oral gavage every day for 14 consecutive days. Nontreated normal and diabetic rats were maintained in similar conditions as the treated rats. Rats were sacrificed on day 15 for testes histology, androgen hormone levels, fertility study (implantation sites), and proteomics analysis.

### 2.3. Testes Histology

Testes were removed and fixed in a Bouin's Solution overnight. The testes were then dehydrated using alcohol and embedded in paraffin wax. Testes samples were sectioned at 5 *μ*m in thickness and stained using Mallory staining. Testicular spermatogenesis was observed under light microscope.

### 2.4. Androgen Hormone Analyses (LH, FSH and Testosterone)

Blood samples were collected through cardiac puncture for the estimation of serum LH, FSH, and testosterone levels using a commercial kit as per manufacturer's instructions (Testosterone EIA, LH, and FSH kit by Cayman Chemical, Michigan, USA) [7].

### 2.5. Number of Implantation Sites

Each male rat from the six experimental groups was kept in a different cage together with two oestrous female rats. Healthy female rats were used in this study and made receptive for sexual activity by subcutaneous injections of 20 *μ*g/rat of estradiol benzoate 52 h prior to mating and 1 mg/rat of progesterone 4 h earlier before mating. The males were allowed to be in the libido box for 10 min before the test for marking purposes [22]. Both males and females were allowed to mate for 7 days. Vaginal smears were performed to determine the gestation day. Pregnant female rats were later separated and kept for 16 days before being sacrificed, and the number of implantation sites in the uteri was recorded [23].

### 2.6. Sperm Proteomics Analysis

The sperm protein samples used in this study were from the three control groups and the best GPAE treatment dose group (450 mg/kg). Three biological replicates from each group were chosen for this study. The protein extraction was prepared as described in Yunianto et al. [15] with modifications. Sperm samples were harvested from the caudal epididymis. Sperms were allowed to “swim up” in Biggers–Whitten–Whittingham medium [24] for 30 min at 37°C in 5% CO_2_ incubator. These sperm samples were centrifuged and lysed with lysis buffer. Sodium dodecyl sulphate gel 12.5% was prepared for the electrophoresis process, which was done at the voltage of 75 V. Subsequently, protein digestion was carried out using in-gel digestion method as described by [25]. A total of 100 *μ*g of protein lysate was incubated with dithiotreitol and iodoacetamide for reduction and alkylation steps, followed by overnight trypsinisation by adding 6 ng/*μ*L trypsin (PROMEGA Gold, USA).

The peptide samples were then submitted into an LC system (Dionex 3000 Ultimate RSLCnano) coupled to an LTQ Orbitrap Fusion mass spectrometer (Thermo Fisher, Bremen, Germany). Digested samples (1 *μ*L) were injected into a reverse phase column and eluted with a flow rate of 300 nL/min. Data were acquired in data-dependent mode. Precursors with an assigned monoisotopic* m/z* and charged 2–7 were further analysed. All precursors were filtered using a 20-s dynamic exclusion window with intensity threshold of 5000. The MS/MS spectra analysis was performed by using rapid scan rate and maximum injection time of 250 ms.

The LC-MS/MS data were analysed using MaxQuant software (version 1.5.3.30) as described by Iovinella et al. [26] and searched against the* Rattus norvegicus* protein sequences obtained from Uniprot database (proteome ID: UP000002494, accessed on February 2016). Peptide spectrum match and protein identification were filtered using a target-decoy approach with a false discover rate of 1%. The second peptide feature was enabled. The label-free quantification (LFQ) of protein was done using the MaxLFQ algorithm integrated in the MaxQuant software. Other MaxQuant settings were at default. All the information was reported in the “proteinGroups” output file, containing the full list of identified and quantified proteins.

The data were further analysed using Perseus software (version 1.5.4.1). The “proteinGroups” output file produced by MaxQuant was analysed. The hits to the reverse database, contaminants, and proteins identified with modified peptides were eliminated. Then, the LFQ intensity ratios were transformed by log_2_. Missing values were computed by drawing random numbers from a normal distribution to stimulate signals from low abundant proteins, using the default parameters [17]. The value of log_2_ (LFQ ratios) for each sample was then statistically analysed. Proteins identified were then annotated by using BLAST2GO (https://www.blast2go.com/) and WEGO analyses (https://biodb.swu.edu.cn/cgi-bin/wego/index.pl).

### 2.7. Statistical Analysis

Data were displayed as mean ± standard error of mean. The statistical analysis was performed in SPSS 22.0, using one-way analysis of variance (ANOVA) with a* p* value <0.05 considered statistically significant. For the proteomics analysis, protein LFQ intensities were compared using Perseus software (version 1.5.4.1) (one-way ANOVA,* p* < 0.05).

## 3. Results

### 3.1. Androgen Hormone Were Elevated after 14 Days of GPAE Treatment

Hormone levels for testosterone, LH, and FSH are shown in Table 1. On the basis of the results, all three hormones decreased in the diabetic rat group compared with the normal group. For example, the normal group displayed a higher level of testosterone (0.757 ± 0.014 ng/mL) compared with the negative control (0.151 ± 0.010 ng/mL). Similarly, both LH and FSH were lower in the diabetic rats (0.017 ± 0.002 ng/mL and 0.020 ± 0.008 ng/mL, respectively) compared with the normal group (0.042 ± 0.002 ng/mL and 0.037 ± 0.008 ng/mL, respectively). Furthermore, the use of metformin did not improve the levels of hormones in comparison with the diabetic group. Conversely, all GPAE treatment groups (14 days after treatment) showed increasing trends of testosterone, LH, and FSH hormone levels, specifically at the dosage of 450 mg/kg (0.802 ± 0.014 ng/mL, 0.041 ± 0.006 ng/mL, and 0.045 ± 0.008 ng/mL, respectively) (Table 1).

### 3.2. GPAE Treatment Improved Testicular Impairment and Number of Implantation Sites

**Figure 1:**
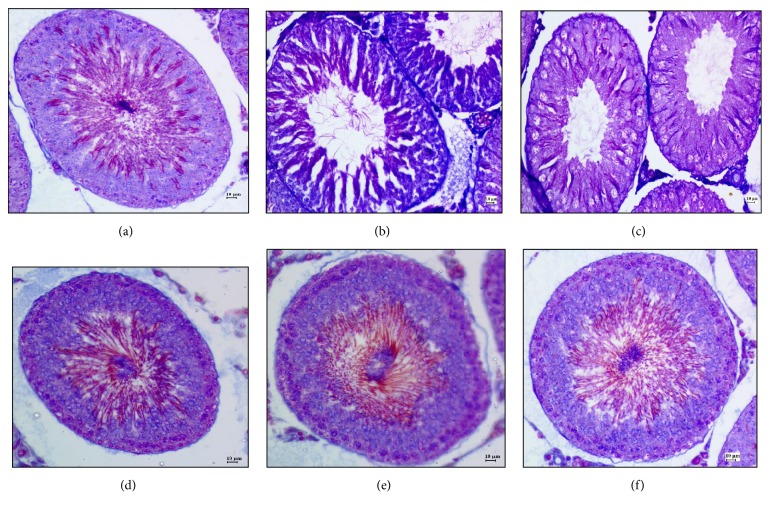
Cross-section of testes for each group at magnification 400×. (a) Normal (nontreated) control, (b) diabetic control (nontreated diabetic), (c) metformin-treated diabetic as well as* Gynura procumbens* aqueous extract (GPAE) treatment; (d) 150 mg/kg, (e) 300 mg/kg, and (f) 450 mg/kg on diabetic rats.

Testicular tissue sections are shown in Figure 1. On the basis of the testes histology, the normal group showed normal spermatogenesis (Figure 1(a)), whereas the diabetic control demonstrated disrupted spermatogenesis as the lumen of seminiferous tubule was almost empty (Figure 1(b)). Similarly, the same histology could be seen in the metformin-treated diabetic control group. Alternatively, the GPAE-treated groups (150, 300, and 450 mg/kg) showed significant changes in the testes histology with the appearance of normal Sertoli and Leydig cells and undisrupted spermatogenesis (Figures 1(d)–1(f)).

**Figure 2:**
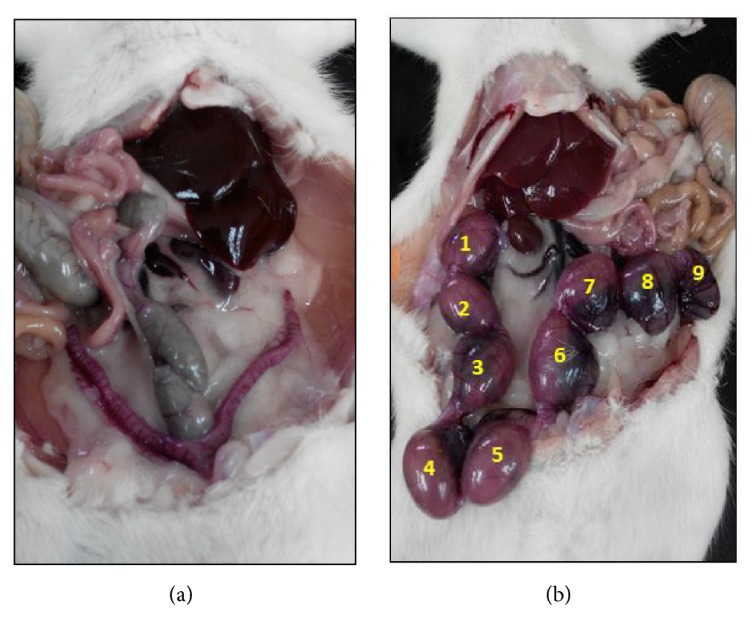
Representative figures of the number of implantation sites after mated with male rats from the (a) diabetic group (nontreated) and (b)* Gynura procumbens* aqueous extract (GPAE) treated group (450 mg/kg).

Furthermore, the number of implantation sites was observed in the uterine horn after 16 days of gestation (Table 2, Figure 2). The number of implantation sites in the normal control was 10 compared with none in the diabetic control rats. For the metformin-treated diabetic group, the number of implantation sites was still low (three sites). Conversely, the implantation sites in the females copulated with GPAE-treated groups increased significantly in the range of 7 to 11 sites, and the highest number was observed in the 450 mg/kg dosage group (Table 2).

### 3.3. Shotgun Proteomics Identified Proteins Related to Various Biological Processes

**Figure 3:**
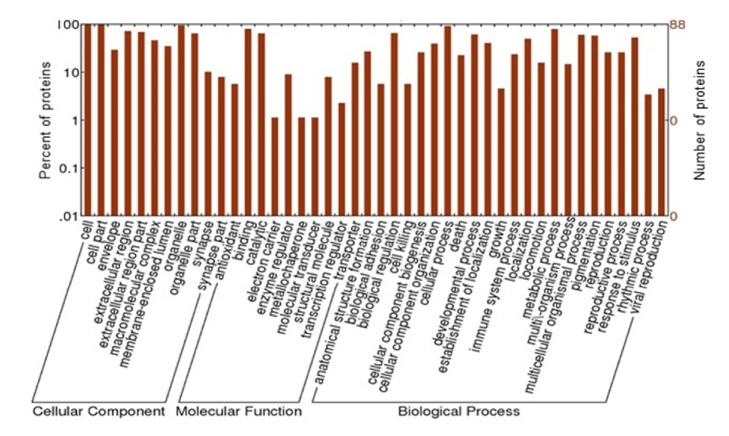
Classification of 88 sperm proteins by gene ontology: cellular component, molecular function, and biological process.

To study the effect of GPAE on the sperm proteins, a shotgun proteomics approach was performed. The 450 mg/kg dose group was used, because it was the most effective treatment compared with the other three control groups. A total of 666 proteins were identified in the first initial run (data not shown) and through a stringent search using MaxQuant software, a total of 88 proteins were found in all groups (Supplementary Table S1). For better insight of the identified proteins, they were annotated using Blast2GO software and classified into cellular component, biological process, and molecular function (Figure 3).

Most of the identified proteins in the biological processes played roles in the metabolic (79.5%), developmental (61.4%), biological regulation (65.9%), and reproduction (26.1%) (Figure 3). Furthermore, the most common molecular activities of the identified proteins were binding (80.7%), catalytic (64.8%), and transporter activities (15.9%) (Figure 3). On the basis of the results, the identified proteins manifested diverse biological roles and molecular activities, specifically in the metabolic pathway.

The 88 identified proteins were then classified according to their biological functions. All proteins were categorised into four general groupings: reproduction, sperm development, locomotion, and sperm metabolic process (Table 3). Fifteen proteins were found to play some roles in reproduction, including cysteine-rich secretory protein-1, zona pellucida binding protein, and outer dense fibre protein. Meanwhile, 10 proteins were involved in the sperm developmental process including carboxylic ester hydrolase, enolase, and lipocalin-5 (Table 3). Eight proteins including tubulin, l-lactate dehydrogenase, and phosphoglycerate kinase were categorised in sperm locomotion (Table 3). Many of the proteins (55) were involved in the metabolic process (Table 3), which is an important event in the living body, particularly in sperm. A large amount of energy is needed for sperm motility and fertility. The metabolic process includes TCA cycle, glycolysis, purine and reductase metabolism. Several proteins involved in these processes were found in this study including glyceraldehyde-3-phosphate, pyruvate dehydrogenase, and glucose-6-phosphate (Table 3).

### 3.4. Differentially Expressed Proteins Highlight GPAE Effect on Spermatogenic Proteins

**Figure 4:**
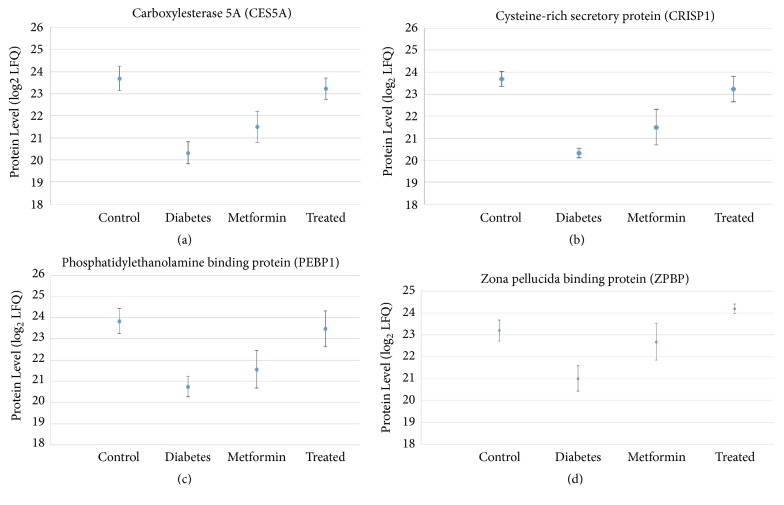
Four significantly expressed proteins in* Gynura procumbens* aqueous extract (GPAE) treatment compared to the diabetic nontreated group. These proteins including carboxylesterase (CES5A) (a), cysteine-rich secretory protein 1 (CRISP1) (b), phosphatidylethanolamine-binding protein1 (PEBP1) (c), and zona pellucida binding protein (ZPBP) (d) are related to sperm development and sperm-egg interaction.

Quantification analysis was performed using MaxQuant software to compare protein levels between the experimental groups, yielding four statistically significant (*p* < 0.05) protein changes between nontreated diabetic rats and GPAE-treated diabetic rats (Figure 4). These proteins were carboxylesterase (CES5A), cysteine-rich secretory protein 1 (CRISP1), phosphatidylethanolamine-binding protein 1 (PEBP1), and zona pellucida binding protein (ZPBP) (Figures 4(a)–4(d)).

CES5A protein level significantly increased in GPAE-treated groups (23.21 ± 0.48) compared with both diabetic and metformin-treated diabetic control, which was at 20.42 ± 0.49 and 22.60 ± 0.77, respectively. The protein level was matched with the normal control at 23.62 ± 0.54. Metformin-treated diabetic control showed a slight increase of protein level compared with diabetic control; however, the level was not significant. For CRISP1, GPAE-treated groups showed a significant increase of protein level (23.24 ± 0.58) compared with both diabetic (20.32 ± 0.22) and metformin-treated diabetic control (21.50 ± 0.81). The third protein found to be significantly expressed after treatment with GPAE was PEBP1. This protein was at a higher level in GPAE-treated groups (23.48 ± 0.84) compared with diabetic and metformin-treated diabetic control. The protein level matched with normal control (23.84 ± 0.66). Both diabetic and metformin-treated diabetic control showed lower protein level at 20.75 ± 0.48 and 21.57 ± 0.89, respectively. In addition, protein level of ZPBP was the highest in GPAE-treated groups at 24.19 ± 0.21, compared with diabetic (21.00 ± 0.59) and metformin-treated diabetic control (22.67 ± 0.21). All these proteins were highly expressed in both normal control as well as 450 mg/kg dose GPAE-treated group, in comparison with the nontreated diabetic rats. Although the average protein levels could be higher for metformin-treated diabetic rats compared with nontreated diabetic rats, most levels were not significantly different and not as high as either or both normal control and GPAE-treated rats (Figure 4).

## 4. Discussion

Diabetes mellitus has always been linked to reproductive dysfunction, mainly in the male reproductive system. Many studies have revealed that diabetes mellitus can affect spermatogenesis and steroidogenesis [20, 27]. In this study, the impact of GPAE herbal treatment on diabetic rats was assessed in regard to the androgen hormone levels, testes histology, and fertilization (implantation sites) as well as sperm protein level.

Diabetes mellitus has been shown to reduce androgen hormones such as LH, FSH, and testosterone [28]. LH and FSH are the main regulatory hormones used for the stimulation of steroid hormones including testosterone and gametogenesis in both men and women [4]. In the current study, LH, FSH, and testosterone levels were significantly elevated in the GPAE-treated groups compared with the diabetic control (nontreated diabetic) group (Table 1). It appears that the GPAE treatment significantly increases the androgen hormone levels to match the normal control group level. The role of FSH is to stimulate Sertoli cells in testes to provide nutrients for sperm throughout the spermatogenesis process [4], while LH is responsible for functional Leydig cells and testosterone production [28]. The increase of both LH and FSH levels (Table 1) as well as reduced hyperglycaemia in GPAE-treated rats may repair the Leydig cells, thus, increasing the testosterone level. As a consequence, a functional male reproductive system can be regenerated, assisting spermatogenesis and the testicular structure regeneration [29].

Evidently, the testes histology of the GPAE-treated group has been improved with Sertoli and Leydig cells regenerated and the sperm in the lumen restored (Figures 1(d)–1(f)) compared with nontreated diabetic rats (Figure 1(b)). This is consistent with our proteomics finding which has identified the increase of CES5A protein level in diabetic rats after GPAE treatment (Figure 4(a)). CES5A is known to be expressed in the corpus and caudal epididymis, as well as being secreted in the lumen [30]. It was reported that the regulation of the lipid environment by CES5A in the epididymal lumen is crucial for sperm maturation and storage [30]. Furthermore, another protein known as PEBP1 was significantly increased in the GPAE-treated group compared with the nontreated diabetic rats (Figure 4(c)). PEBP1 is involved in sperm development and capacitation [31]. It also plays a role as a decapacitation factor (DF) receptor [31] which is vital in the regulation of sperm function. Hence, the higher CES5A and PEBP1 levels in GPAE-treated rats may suggest the profertility impact of the herbal treatment on diabetic rats, particularly with regard to the regeneration of testes structure and spermatogenesis.

GPAE treatment was also shown to significantly increase sperm quality including sperm count, motility, viability, and morphology compared with the diabetic control group [14]. As such, this has also improved the fertility of the male rats as evidence by the significant number of implantation sites (7 to 11 sites) in fertilized female rats compared with the diabetic control group (Table 2). Congruently, a few differentially expressed proteins identified in this study were involved in fertilization process. For example, ZPBP was found to be significantly expressed in GPAE-treated group compared with diabetic and metformin-treated diabetic control groups (Figure 4(d)). ZPBP is localised to the acrosome region and is important for the binding of sperm to the zona pellucida of the oocyte [32, 33]. The binding of sperm to zona pellucida membrane is one of the most crucial parts in sperm-egg interaction which leads to a successful penetration. Another important protein for fertilization is CRISP1 which was also significantly expressed upon GPAE treatment (Figure 4(b)). CRISP1 was reported to participate in sperm-egg fusion through the interaction with complementary sites on the surface of the egg [34, 35]. The increase of both ZPBP and CRISP1 in GPAE-treated diabetic rats implies that the herbal treatment was effective in increasing fertility in diabetic rats of which the changes can be implicated at the molecular protein level.

In contrast, metformin treatment demonstrates no improvement in most of our fertility tests (Figure 1, Tables 1 and 2). Previous studies showed that the drug was able to reduce the diabetic rat's blood sugar [12, 14], signifying that metformin was only effective against diabetes. This is further corroborated by the observed levels of androgen hormones (LH, FSH, and testosterone) which were not significantly higher than the nontreated diabetic rats (Table 2). Without improvement in these hormones, the repair and regeneration processes may not be able to occur, leading to degenerated Leydig cells and defective testes histology (Figure 1(c)). Consequently, the fertility of the male rats was not effectively recovered, as observed by the low number of implantation sites (only three sites) in female rats (Table 2).

## 5. Conclusion

In conclusion, the results of this study provide evidence that GPAE may improve androgen hormone levels, testicular regeneration, and fertility (number of implantation sites). Proteomic analysis further implies that GPAE improved the fertility of diabetic male rats via increasing protein expression related to sperm maturation and sperm-egg interaction. Thus, GP may have potential as an antihyperglycaemia and profertility agent for diabetic patients. Further studies are needed to develop the herbal extract as an alternative medication to treat diabetes and fertility problems.

## Figures and Tables

**Table 1:** The effect of *G. procumbens* treatment on androgen hormone levels (luteinizing, follicle stimulating, and testosterone hormones) of diabetic male rats. Different letters beside the numbers suggest significantly different according to one-way ANOVA (p < 0.05).

Group	Testosterone hormone (ng/mL)	Luteinizing hormone (ng/mL)	Follicle-stimulating hormone (ng/mL)
Normal	0.757 ± 0.014^b,c^	0.042 ± 0.002^b,c^	0.037 ± 0.008^b,c^
Diabetes	0.151 ± 0.010	0.017 ± 0.002	0.020 ± 0.008
Metformin	0.179 ± 0.009	0.021 ± 0.002	0.021 ± 0.004^b^
150 mg/kg	0.754 ± 0.008^b,c^	0.031 ± 0.003^b,c^	0.027 ± 0.007^b,c^
300 mg/kg	0.746 ± 0.009^b,c^	0.032 ± 0.006^b,c^	0.033 ± 0.008^b,c^
450 mg/kg	0.802 ± 0.014^a,b,c^	0.041 ± 0.006^b,c^	0.045 ± 0.008^a,b,c^

^a^Significantly increased compared to normal control (p<0.05); ^b^significantly increased compared to diabetic control (p<0.05); ^c^significantly increased compared to metformin-treated diabetic control (p<0.05).

**Table 2:** The effect of *G. procumbens* aqueous extract treatment on the number of female implantation sites upon copulation with the treated group male rats. Different letters beside the numbers suggest significant difference according to one-way ANOVA (p < 0.05).

Group	Number of implantation sites
Normal	10 ± 0.62
Diabetes	00 ± 0.29
Metformin	03 ± 1.06^a^
150 mg/kg	07 ± 0.49^a,b^
300 mg/kg	06 ± 0.36^a,b^
450 mg/kg	11 ± 0.57^a,b^

^a^Significantly increased compared to diabetic control (p<0.05); ^b^significantly increased compared to metformin-treated diabetic control (p<0.05).

**Table 3:** Classification of 88 identified sperm proteins according to their biological processes. All proteins have Q value of 0 (Supplementary Table 1).

**No**	**ID**	**Protein names**	**Peptides**	**Sequence Coverage**	**Molecular Weight**	**Score**	**MS/MS Count**
**Proteins involved in reproduction**							

1	P12020	Cysteine-rich secretory protein 1 (32 kDa epididymal protein) (Acidic epididymal glycoprotein) (Protein D) (Protein E) (Protein IV) (Sialoprotein) (Sperm-coating glycoprotein) (SCP)	8	32.1	27.847	89.627	78
2	P31044	Phosphatidylethanolamine-binding protein 1 (PEBP-1) (23 kDa morphine-binding protein) (HCNPpp) (P23K) [Cleaved into: Hippocampal cholinergic neurostimulating peptide (HCNP)]	8	73.8	20.801	314.36	111
3	Q6AXU2	Zona pellucida binding protein, isoform CRA_b (Zona pellucida-binding protein)	17	40.3	45.129	323.31	188
4	A0A0H2UHA4	Mitochondria-eating protein	12	17.4	67.794	145.83	37
5	M0R4L7	Histone H2B	6	48.4	13.91	85.052	41
6	A0A0H2UHP1	Retinal dehydrogenase 1	17	34	56.589	323.31	146
7	D3ZE94	Outer dense fiber of sperm tails 3 (Outer dense fiber of sperm tails 3 (Predicted))	13	53.1	27.879	135.33	72
8	D4ACV3	Histone H2A	6	43.7	13.645	323.31	52
9	M0R660	Glyceraldehyde-3-phosphate dehydrogenase (EC 1.2.1.12)	13	38.1	35.783	323.31	83
10	P18163	Long-chain-fatty-acid--CoA ligase 1 (EC 6.2.1.3) (Long-chain acyl-CoA synthetase 1) (LACS 1) (Long-chain-fatty-acid--CoA ligase, liver isozyme)	19	37.1	78.178	323.31	109
11	P55063	Heat shock 70 kDa protein 1-like (Heat shock 70 kDa protein 1L) (Heat shock 70 kDa protein 3) (HSP70.3)	27	43.7	70.548	323.31	202
12	G3V7X0	Outer dense fiber of sperm tails 2, isoform CRA_e (Outer dense fiber protein 2)	60	65	81.346	323.31	360
13	Q4KLL5	Ropporin-1 (Rhophilin-associated protein 1)	10	58.5	23.96	322.26	67
14	Q6AXR4	Beta-hexosaminidase subunit beta (EC 3.2.1.52) (Beta-N-acetylhexosaminidase subunit beta) (Hexosaminidase subunit B) (N-acetyl-beta-glucosaminidase subunit beta)	18	26.6	61.527	198.66	66
15	Q6P502	T-complex protein 1 subunit gamma (TCP-1-gamma) (CCT-gamma)	11	30.1	60.646	186.2	52

**Proteins involved in sperm development process**							

1	F1M786	Carboxylic ester hydrolase (EC 3.1.1.-)	14	22.5	61.398	323.31	107
2	P06911	Epididymal-specific lipocalin-5 (Androgen-dependent epididymal 18.5 kDa protein) (Epididymal retinoic acid-binding protein) (E-RABP) (Epididymal secretory protein I) (ESP-I) [Cleaved into: Epididymal-specific lipocalin-5, B form; Epididymal-specific lipocalin-5, C form]	9	58	20.67	235.53	89
3	A0A0G2K3K2	Actin, cytoplasmic 1	22	62.7	41.792	323.31	457
4	G3V7J0	Aldehyde dehydrogenase family 6, subfamily A1, isoform CRA_b (Methylmalonate-semialdehyde dehydrogenase [acylating], mitochondrial)	12	35.1	57.747	205	70
5	G3V9D8	Carboxylic ester hydrolase (EC 3.1.1.-)	11	24.8	62.17	323.31	104
6	P04905	Glutathione S-transferase Mu 1 (EC 2.5.1.18) (GST 3-3) (GSTM1-1) (Glutathione S-transferase Yb-1) (GST Yb1)	12	55.5	25.914	195.33	56
7	P11951	Cytochrome c oxidase subunit 6C-2 (Cytochrome c oxidase polypeptide VIc-2)	3	36.8	8.4548	38.138	31
8	Q6YFQ1	Cytochrome c oxidase subunit 6B2 (Cytochrome c oxidase subunit VIb isoform 2) (COX VIb-2) (Cytochrome c oxidase subunit VIb, testis-specific isoform)	4	46.6	10.472	28.904	28
9	P10715	Cytochrome c, testis-specific	13	77.1	11.742	323.31	114
10	Q5BJ93	Enolase 1, (Alpha) (RCG31027, isoform CRA_a)	35	77.2	47.127	323.31	472

**Proteins involved in locomotion**							

1	G3V7C6	Tubulin beta chain	44	81.6	61.186	323.31	1073
2	Q6AYX2	L-lactate dehydrogenase (EC 1.1.1.27)	22	72.3	35.712	323.31	195
3	B5DEN4	L-lactate dehydrogenase (EC 1.1.1.27)	8	28	36.45	323.31	47
4	P68370	Tubulin alpha-1A chain (Alpha-tubulin 1) (Tubulin alpha-1 chain) [Cleaved into: Detyrosinated tubulin alpha-1A chain]	21	47	50.135	20.5	23
5	Q5XIV1	Phosphoglycerate kinase (EC 2.7.2.3)	36	75.8	45.01	323.31	545
6	Q6AYM2	Tektin-2 (Tektin-t) (Testicular tektin)	6	17	50.296	54.918	23
7	P63269	Actin, gamma-enteric smooth muscle (Alpha-actin-3) (Gamma-2-actin) (Smooth muscle gamma-actin) [Cleaved into: Actin, gamma-enteric smooth muscle, intermediate form]	20	60.6	41.876	173.1	38
8	Q68FR8	Tubulin alpha-3 chain (Alpha-tubulin 3) [Cleaved into: Detyrosinated tubulin alpha-3 chain]	24	54.7	49.959	323.31	363

**Proteins involved in metabolism**							

1	G3V8X9	Serine (or cysteine) peptidase inhibitor, clade A (alpha-1 antiproteinase, antitrypsin), member 16 (Serine proteinase inhibitor HongrES1)	24	48.2	47.181	323.31	192
2	M0R8P3	Calcium-binding tyrosine phosphorylation-regulated (RCG24939, isoform CRA_c)	7	30.8	43.044	131.18	57
3	O88767	Protein/nucleic acid deglycase DJ-1 (EC 3.1.2.-) (EC 3.5.1.-) (EC 3.5.1.124) (Contraception-associated protein 1) (Protein CAP1) (Fertility protein SP22) (Maillard deglycase) (Parkinson disease protein 7 homolog) (Parkinsonism-associated deglycase) (Protein DJ-1) (DJ-1)	8	33.9	19.974	173.44	77
4	P00507	Aspartate aminotransferase, mitochondrial (mAspAT) (EC 2.6.1.1) (EC 2.6.1.7) (Fatty acid-binding protein) (FABP-1) (Glutamate oxaloacetate transaminase 2) (Kynurenine aminotransferase 4) (Kynurenine aminotransferase IV) (Kynurenine--oxoglutarate transaminase 4) (Kynurenine--oxoglutarate transaminase IV) (Plasma membrane-associated fatty acid-binding protein) (FABPpm) (Transaminase A)	14	44.7	47.314	218.74	98
5	P02091	Hemoglobin subunit beta-1 (Beta-1-globin) (Hemoglobin beta chain, major-form) (Hemoglobin beta-1 chain)	21	70.7	15.979	323.31	481
6	P02770	Serum albumin	52	74.5	68.73	323.31	688
7	P04636	Malate dehydrogenase, mitochondrial (EC 1.1.1.37)	11	53.8	35.683	130.31	33
8	P05065	Fructose-bisphosphate aldolase A (EC 4.1.2.13) (Muscle-type aldolase)	19	59.3	39.351	323.31	127
9	P06761	Endoplasmic reticulum chaperone BiP (EC 3.6.4.10) (78 kDa glucose-regulated protein) (GRP-78) (Binding-immunoglobulin protein) (BiP) (Heat shock protein 70 family protein 5) (HSP70 family protein 5) (Heat shock protein family A member 5) (Immunoglobulin heavy chain-binding protein) (Steroidogenesis-activator polypeptide)	21	33	72.346	323.31	184
10	P09606	Glutamine synthetase (GS) (EC 6.3.1.2) (Glutamate decarboxylase) (EC 4.1.1.15) (Glutamate--ammonia ligase)	10	30	42.267	323.31	156
11	P10760	Adenosylhomocysteinase (AdoHcyase) (EC 3.3.1.1) (S-adenosyl-L-homocysteine hydrolase)	3	6.9	47.538	122.35	23
12	P10818	Cytochrome c oxidase subunit 6A1, mitochondrial (Cytochrome c oxidase polypeptide VIa-liver)	5	63.1	12.301	63.576	48
13	P10860	Glutamate dehydrogenase 1, mitochondrial (GDH 1) (EC 1.4.1.3) (Memory-related gene 2 protein) (MRG-2)	9	17	61.415	177.81	83
14	P11517	Hemoglobin subunit beta-2 (Beta-2-globin) (Hemoglobin beta chain, minor-form) (Hemoglobin beta-2 chain)	18	64.6	15.982	323.31	158
15	P11980	Pyruvate kinase PKM (EC 2.7.1.40) (Pyruvate kinase muscle isozyme)	15	40.1	57.817	323.31	84
16	P12346	Serotransferrin (Transferrin) (Beta-1 metal-binding globulin) (Liver regeneration-related protein LRRG03) (Siderophilin)	17	28.4	76.394	317.85	139
17	P16290	Phosphoglycerate mutase 2 (EC 5.4.2.11) (EC 5.4.2.4) (BPG-dependent PGAM 2) (Muscle-specific phosphoglycerate mutase) (Phosphoglycerate mutase isozyme M) (PGAM-M)	16	71.1	28.755	323.31	155
18	P19804	Nucleoside diphosphate kinase B (NDK B) (NDP kinase B) (EC 2.7.4.6) (Histidine protein kinase NDKB) (EC 2.7.13.3) (P18)	3	38.8	17.283	55.113	20
19	P20760	Ig gamma-2A chain C region	10	32.6	35.185	172.46	82
20	P32551	Cytochrome b-c1 complex subunit 2, mitochondrial (Complex III subunit 2) (Core protein II) (Ubiquinol-cytochrome-c reductase complex core protein 2)	4	20.1	48.396	323.31	29
21	P46462	Transitional endoplasmic reticulum ATPase (TER ATPase) (EC 3.6.4.6) (15S Mg(2+)-ATPase p97 subunit) (Valosin-containing protein) (VCP)	23	41.8	89.348	323.31	136
22	Q4KLZ6	Triokinase/FMN cyclase (Bifunctional ATP-dependent dihydroxyacetone kinase/FAD-AMP lyase (cyclizing)) [Includes: ATP-dependent dihydroxyacetone kinase (DHA kinase) (EC 2.7.1.28) (EC 2.7.1.29) (Glycerone kinase) (Triokinase) (Triose kinase); FAD-AMP lyase (cyclizing) (EC 4.6.1.15) (FAD-AMP lyase (cyclic FMN forming)) (FMN cyclase)]	8	27.7	59.443	202.32	59
23	Q4QR77	Protein FAM166A	4	18.4	36.993	102.93	31
24	Q4V8H5	Aspartyl aminopeptidase (Aspartyl aminopeptidase, isoform CRA_c)	9	24.2	52.555	323.31	60
25	Q4V8P4	Rsb-66 protein (Rsb-66 protein, isoform CRA_b) (Sperm acrosome-associated 9)	3	26.2	19.465	323.31	56
26	Q5RK28	Normal mucosa of esophagus-specific gene 1 protein	9	83.1	9.5981	275.93	78
27	Q5XI62	Protein MENT (Methylated in normal thymocytes protein)	10	45.3	37.64	246.1	65
28	Q6AXN7	5'-nucleotidase, cytosolic IB	21	39.5	64.922	323.31	134
29	Q6AXX6	Redox-regulatory protein FAM213A (Peroxiredoxin-like 2 activated in M-CSF stimulated monocytes) (Protein PAMM) (Sperm head protein 1)	14	50.7	25.763	240.02	79
30	Q6AY07	Fructose-bisphosphate aldolase (EC 4.1.2.13)	20	56.6	39.491	323.31	220
31	Q6AY30	Saccharopine dehydrogenase-like oxidoreductase (EC 1.-.-.-)	12	38.5	47.088	204.86	50
32	Q6P6V0	Glucose-6-phosphate isomerase (GPI) (EC 5.3.1.9) (Autocrine motility factor) (AMF) (Neuroleukin) (NLK) (Phosphoglucose isomerase) (PGI) (Phosphohexose isomerase) (PHI)	14	38.7	62.826	323.31	73
33	Q6P762	Alpha-mannosidase (EC 3.2.1.-)	23	29.7	114.33	323.31	113
34	Q8VI04	Isoaspartyl peptidase/L-asparaginase (EC 3.4.19.5) (EC 3.5.1.1) (Asparaginase-like protein 1) (Asparaginase-like sperm autoantigen) (Beta-aspartyl-peptidase) (Glial asparaginase) (Isoaspartyl dipeptidase) (L-asparagine amidohydrolase) [Cleaved into: Isoaspartyl peptidase/L-asparaginase alpha chain; Isoaspartyl peptidase/L-asparaginase beta chain]	14	49.8	34.41	323.31	45
35	A0A0G2KAM3	Pyruvate dehydrogenase E1 component subunit beta, mitochondrial	4	11.1	46.192	272.59	17
36	A0A0G2JSJ3	Solute carrier family 2 (Facilitated glucose transporter), member 3 (Solute carrier family 2, facilitated glucose transporter member 3-like)	5	14	53.562	151.67	31
37	A0A0G2JSJ8	Fucosidase, alpha-L-1, tissue, isoform CRA_a (Tissue alpha-L-fucosidase)	5	15.4	53.472	286.49	63
38	A0A0G2JSV6	Globin c2 (Hemoglobin alpha, adult chain 2) (RCG34342, isoform CRA_a)	17	69.7	15.284	323.31	587
39	A0A0G2JSZ5	Protein disulfide-isomerase A6 (RCG62282, isoform CRA_a)	6	17.3	48.76	96.801	26
40	A0A0G2JTW9	Hemoglobin, beta adult major chain	10	70.7	15.988	138.77	32
41	G3V8Q6	cAMP-dependent protein kinase type II-alpha regulatory subunit	6	29.9	45.48	173.45	23
42	D4AA52	Alpha-1-inhibitor III	8	6.3	163.6	53.696	34
43	A0A0H2UHE1	Succinate--CoA ligase [ADP/GDP-forming] subunit alpha, mitochondrial (EC 6.2.1.4) (EC 6.2.1.5) (Succinyl-CoA synthetase subunit alpha) (SCS-alpha)	6	21.1	37.559	218	38
44	A0A0H2UHM5	Protein disulfide-isomerase (EC 5.3.4.1)	22	49	57.078	272.63	83
45	B0K020	CDGSH iron-sulfur domain-containing protein 1 (MitoNEET)	6	57.4	12.097	95.47	82
46	B1H216	Globin c3 (Hemoglobin alpha, adult chain 2) (RCG34636, isoform CRA_a)	16	69.7	15.328	318.53	41
47	B6DYP8	Glutathione S-transferase (EC 2.5.1.18)	9	55.2	25.319	150.96	69
48	D3Z9F9	Similar to RIKEN cDNA 4930540L03 (Predicted) (Sperm acrosome-associated 1)	5	25.4	37.335	302.34	54
49	D3ZLJ6	Amine oxidase (EC 1.4.3.-)	5	11.7	71.083	31.503	33
50	D3ZUM4	Beta-galactosidase (EC 3.2.1.23)	21	33.5	73.227	224.16	110
51	D4A4R7	RCG21015, isoform CRA_a (Serine (or cysteine) peptidase inhibitor, clade A, member 1F)	4	16.5	46.985	33.071	36
52	F1LML2	Polyubiquitin-C	8	90.2	91.072	323.31	98
53	F1LN88	Aldehyde dehydrogenase, mitochondrial (RCG21519, isoform CRA_a)	16	44.7	56.516	323.31	103
54	F1LP05	ATP synthase subunit alpha	17	25.9	59.812	303.31	85
55	G3V6D3	ATP synthase subunit beta (EC 3.6.3.14)	21	57.8	56.344	323.31	153

## Data Availability

All data used to support the findings of this study are included within the supplementary information file.

## Supplementary Materials

Supplementary Materials Supplementary Table 1. A total of 88 sperm proteins identified using MaxQuant and Perseus analysis.
