# Supplementary material for: Gynura procumbens Improved Fertility of Diabetic Rats: Preliminary Study of Sperm Proteomic
Source: Evid Based Complement Alternat Med. 2018 Sep 30;2018:9201539. doi: 10.1155/2018/9201539 (PMC6186343; doi:10.1155/2018/9201539)
Supplement: Supplementary Materials — Supplementary Table 1. A total of 88 sperm proteins identified using MaxQuant and Perseus analysis. [file 9201539.f1.doc]

| ID  Supplementary table 1. 88 total sperm protein identified using MaxQuant and Purseus analysis | Protein names | Peptides | Sequence Coverage | Molecular Weight | Q value | LFQ intensity  CONTROL | LFQ intensity  DIABETES | LFQ intensity  METFORMIN | LFQ intensity  TREATED 450 | Score | MS/MS Count |
| --- | --- | --- | --- | --- | --- | --- | --- | --- | --- | --- | --- |
| A0A0G2KAM3 | Pyruvate dehydrogenase E1 component subunit beta, mitochondrial | 4 | 11.1 | 46.192 | 0 | 20.493 | 20.685 | 22.370 | 20.272 | 272.59 | 17 |
| F1M786 | Carboxylic ester hydrolase (EC 3.1.1.-) | 14 | 22.5 | 61.398 | 0 | 23.616 | 20.421 | 22.608 | 23.205 | 323.31 | 107 |
| P12020 | Cysteine-rich secretory protein 1 (32 kDa epididymal protein) (Acidic epididymal glycoprotein) (Protein D) (Protein E) (Protein IV) (Sialoprotein) (Sperm-coating glycoprotein) (SCP) | 8 | 32.1 | 27.847 | 0 | 23.693 | 20.321 | 21.500 | 23.237 | 89.627 | 78 |
| P31044 | Phosphatidylethanolamine-binding protein 1 (PEBP-1) (23 kDa morphine-binding protein) (HCNPpp) (P23K) [Cleaved into: Hippocampal cholinergic neurostimulating peptide (HCNP)] | 8 | 73.8 | 20.801 | 0 | 23.841 | 20.748 | 21.565 | 23.482 | 314.36 | 111 |
| Q6AXU2 | Zona pellucida binding protein, isoform CRA_b (Zona pellucida-binding protein) | 17 | 40.3 | 45.129 | 0 | 23.195 | 20.997 | 22.673 | 24.189 | 323.31 | 188 |
| P06911 | Epididymal-specific lipocalin-5 (Androgen-dependent epididymal 18.5 kDa protein) (Epididymal retinoic acid-binding protein) (E-RABP) (Epididymal secretory protein I) (ESP-I) [Cleaved into: Epididymal-specific lipocalin-5, B form; Epididymal-specific lipocalin-5, C form] | 9 | 58 | 20.67 | 0 | 23.351 | 21.695 | 23.026 | 24.010 | 235.53 | 89 |
| A0A0G2JSJ3 | Solute carrier family 2 (Facilitated glucose transporter), member 3 (Solute carrier family 2, facilitated glucose transporter member 3-like) | 5 | 14 | 53.562 | 0 | 21.464 | 20.609 | 21.735 | 21.067 | 151.67 | 31 |
| A0A0G2JSJ8 | Fucosidase, alpha-L-1, tissue, isoform CRA_a (Tissue alpha-L-fucosidase) | 5 | 15.4 | 53.472 | 0 | 22.147 | 20.991 | 21.585 | 22.490 | 286.49 | 63 |
| A0A0G2JSV6 | Globin c2 (Hemoglobin alpha, adult chain 2) (RCG34342, isoform CRA_a) | 17 | 69.7 | 15.284 | 0 | 26.922 | 26.727 | 25.865 | 27.623 | 323.31 | 587 |
| A0A0G2JSZ5 | Protein disulfide-isomerase A6 (RCG62282, isoform CRA_a) | 6 | 17.3 | 48.76 | 0 | 20.775 | 20.608 | 21.043 | 19.970 | 96.801 | 26 |
| A0A0G2JTW9 | Hemoglobin, beta adult major chain | 10 | 70.7 | 15.988 | 0 | 21.551 | 20.186 | 21.138 | 21.180 | 138.77 | 32 |
| M0R4L7 | Histone H2B | 6 | 48.4 | 13.91 | 0 | 21.438 | 21.727 | 21.934 | 21.870 | 85.052 | 41 |
| A0A0G2K3K2 | Actin, cytoplasmic 1 | 22 | 62.7 | 41.792 | 0 | 26.105 | 25.815 | 24.254 | 25.689 | 323.31 | 457 |
| G3V8Q6 | cAMP-dependent protein kinase type II-alpha regulatory subunit | 6 | 29.9 | 45.48 | 0 | 21.832 | 20.479 | 21.520 | 20.563 | 173.45 | 23 |
| D4AA52 | Alpha-1-inhibitor III | 8 | 6.3 | 163.6 | 0 | 20.492 | 20.582 | 20.858 | 20.471 | 53.696 | 34 |
| A0A0H2UHA4 | Mitochondria-eating protein | 12 | 17.4 | 67.794 | 0 | 21.378 | 20.476 | 20.991 | 21.890 | 145.83 | 37 |
| A0A0H2UHE1 | Succinate--CoA ligase [ADP/GDP-forming] subunit alpha, mitochondrial (EC 6.2.1.4) (EC 6.2.1.5) (Succinyl-CoA synthetase subunit alpha) (SCS-alpha) | 6 | 21.1 | 37.559 | 0 | 21.403 | 21.645 | 21.674 | 21.587 | 218 | 38 |
| A0A0H2UHM5 | Protein disulfide-isomerase (EC 5.3.4.1) | 22 | 49 | 57.078 | 0 | 22.620 | 21.547 | 21.510 | 21.760 | 272.63 | 83 |
| A0A0H2UHP1 | Retinal dehydrogenase 1 | 17 | 34 | 56.589 | 0 | 22.987 | 23.460 | 22.564 | 22.739 | 323.31 | 146 |
| B0K020 | CDGSH iron-sulfur domain-containing protein 1 (MitoNEET) | 6 | 57.4 | 12.097 | 0 | 21.610 | 21.516 | 21.920 | 22.730 | 95.47 | 82 |
| B1H216 | Globin c3 (Hemoglobin alpha, adult chain 2) (RCG34636, isoform CRA_a) | 16 | 69.7 | 15.328 | 0 | 23.525 | 21.742 | 21.502 | 25.038 | 318.53 | 41 |
| B5DEN4 | L-lactate dehydrogenase (EC 1.1.1.27) | 8 | 28 | 36.45 | 0 | 21.932 | 21.440 | 22.616 | 20.871 | 323.31 | 47 |
| B6DYP8 | Glutathione S-transferase (EC 2.5.1.18) | 9 | 55.2 | 25.319 | 0 | 22.682 | 22.588 | 21.624 | 22.688 | 150.96 | 69 |
| D3Z9F9 | Similar to RIKEN cDNA 4930540L03 (Predicted) (Sperm acrosome-associated 1) | 5 | 25.4 | 37.335 | 0 | 22.346 | 20.617 | 22.297 | 22.235 | 302.34 | 54 |
| D3ZE94 | Outer dense fiber of sperm tails 3 (Outer dense fiber of sperm tails 3 (Predicted)) | 13 | 53.1 | 27.879 | 0 | 21.354 | 20.990 | 21.695 | 22.512 | 135.33 | 72 |
| D3ZLJ6 | Amine oxidase (EC 1.4.3.-) | 5 | 11.7 | 71.083 | 0 | 20.905 | 21.145 | 20.853 | 20.440 | 31.503 | 33 |
| D3ZUM4 | Beta-galactosidase (EC 3.2.1.23) | 21 | 33.5 | 73.227 | 0 | 22.721 | 21.779 | 22.112 | 22.407 | 224.16 | 110 |
| D4ACV3 | Histone H2A | 6 | 43.7 | 13.645 | 0 | 21.678 | 23.138 | 22.077 | 21.964 | 323.31 | 52 |
| D4A4R7 | RCG21015, isoform CRA_a (Serine (or cysteine) peptidase inhibitor, clade A, member 1F) | 4 | 16.5 | 46.985 | 0 | 21.101 | 20.670 | 21.357 | 21.137 | 33.071 | 36 |
| F1LML2 | Polyubiquitin-C | 8 | 90.2 | 91.072 | 0 | 22.851 | 21.714 | 21.866 | 22.779 | 323.31 | 98 |
| F1LN88 | Aldehyde dehydrogenase, mitochondrial (RCG21519, isoform CRA_a) | 16 | 44.7 | 56.516 | 0 | 22.873 | 22.053 | 22.706 | 22.266 | 323.31 | 103 |
| F1LP05 | ATP synthase subunit alpha | 17 | 25.9 | 59.812 | 0 | 21.007 | 22.489 | 23.751 | 21.375 | 303.31 | 85 |
| G3V6D3 | ATP synthase subunit beta (EC 3.6.3.14) | 21 | 57.8 | 56.344 | 0 | 22.108 | 22.323 | 23.917 | 22.304 | 323.31 | 153 |
| G3V7C6 | Tubulin beta chain | 44 | 81.6 | 61.186 | 0 | 28.183 | 24.069 | 26.341 | 27.984 | 323.31 | 1073 |
| G3V7J0 | Aldehyde dehydrogenase family 6, subfamily A1, isoform CRA_b (Methylmalonate-semialdehyde dehydrogenase [acylating], mitochondrial) | 12 | 35.1 | 57.747 | 0 | 21.967 | 20.974 | 22.063 | 22.160 | 205 | 70 |
| G3V7X0 | Outer dense fiber of sperm tails 2, isoform CRA_e (Outer dense fiber protein 2) | 60 | 65 | 81.346 | 0 | 24.816 | 21.777 | 23.770 | 24.817 | 323.31 | 360 |
| G3V8X9 | Serine (or cysteine) peptidase inhibitor, clade A (alpha-1 antiproteinase, antitrypsin), member 16 (Serine proteinase inhibitor HongrES1) | 24 | 48.2 | 47.181 | 0 | 23.707 | 22.324 | 23.930 | 24.029 | 323.31 | 192 |
| G3V9D8 | Carboxylic ester hydrolase (EC 3.1.1.-) | 11 | 24.8 | 62.17 | 0 | 23.240 | 22.184 | 21.579 | 23.401 | 323.31 | 104 |
| M0R660 | Glyceraldehyde-3-phosphate dehydrogenase (EC 1.2.1.12) | 13 | 38.1 | 35.783 | 0 | 23.063 | 22.767 | 21.506 | 22.452 | 323.31 | 83 |
| M0R8P3 | Calcium-binding tyrosine phosphorylation-regulated (RCG24939, isoform CRA_c) | 7 | 30.8 | 43.044 | 0 | 22.729 | 20.570 | 22.486 | 21.082 | 131.18 | 57 |
| O88767 | Protein/nucleic acid deglycase DJ-1 (EC 3.1.2.-) (EC 3.5.1.-) (EC 3.5.1.124) (Contraception-associated protein 1) (Protein CAP1) (Fertility protein SP22) (Maillard deglycase) (Parkinson disease protein 7 homolog) (Parkinsonism-associated deglycase) (Protein DJ-1) (DJ-1) | 8 | 33.9 | 19.974 | 0 | 23.155 | 21.435 | 21.809 | 23.316 | 173.44 | 77 |
| P00507 | Aspartate aminotransferase, mitochondrial (mAspAT) (EC 2.6.1.1) (EC 2.6.1.7) (Fatty acid-binding protein) (FABP-1) (Glutamate oxaloacetate transaminase 2) (Kynurenine aminotransferase 4) (Kynurenine aminotransferase IV) (Kynurenine--oxoglutarate transaminase 4) (Kynurenine--oxoglutarate transaminase IV) (Plasma membrane-associated fatty acid-binding protein) (FABPpm) (Transaminase A) | 14 | 44.7 | 47.314 | 0 | 22.090 | 22.235 | 22.100 | 22.437 | 218.74 | 98 |
| P02091 | Hemoglobin subunit beta-1 (Beta-1-globin) (Hemoglobin beta chain, major-form) (Hemoglobin beta-1 chain) | 21 | 70.7 | 15.979 | 0 | 26.803 | 25.700 | 25.242 | 26.362 | 323.31 | 481 |
| P02770 | Serum albumin | 52 | 74.5 | 68.73 | 0 | 26.891 | 26.048 | 26.153 | 26.928 | 323.31 | 688 |
| P04636 | Malate dehydrogenase, mitochondrial (EC 1.1.1.37) | 11 | 53.8 | 35.683 | 0 | 21.310 | 20.308 | 21.771 | 21.242 | 130.31 | 33 |
| Q5BJ93 | Enolase 1, (Alpha) (RCG31027, isoform CRA_a) | 35 | 77.2 | 47.127 | 0 | 25.818 | 23.404 | 24.278 | 26.237 | 323.31 | 472 |
| P04905 | Glutathione S-transferase Mu 1 (EC 2.5.1.18) (GST 3-3) (GSTM1-1) (Glutathione S-transferase Yb-1) (GST Yb1) | 12 | 55.5 | 25.914 | 0 | 22.441 | 22.607 | 20.840 | 20.576 | 195.33 | 56 |
| P05065 | Fructose-bisphosphate aldolase A (EC 4.1.2.13) (Muscle-type aldolase) | 19 | 59.3 | 39.351 | 0 | 22.855 | 22.664 | 22.967 | 24.063 | 323.31 | 127 |
| P06761 | Endoplasmic reticulum chaperone BiP (EC 3.6.4.10) (78 kDa glucose-regulated protein) (GRP-78) (Binding-immunoglobulin protein) (BiP) (Heat shock protein 70 family protein 5) (HSP70 family protein 5) (Heat shock protein family A member 5) (Immunoglobulin heavy chain-binding protein) (Steroidogenesis-activator polypeptide) | 21 | 33 | 72.346 | 0 | 23.400 | 22.043 | 22.368 | 23.410 | 323.31 | 184 |
| P09606 | Glutamine synthetase (GS) (EC 6.3.1.2) (Glutamate decarboxylase) (EC 4.1.1.15) (Glutamate--ammonia ligase) | 10 | 30 | 42.267 | 0 | 24.021 | 21.382 | 22.532 | 23.891 | 323.31 | 156 |
| P10715 | Cytochrome c, testis-specific | 13 | 77.1 | 11.742 | 0 | 23.972 | 21.711 | 23.071 | 23.488 | 323.31 | 114 |
| P10760 | Adenosylhomocysteinase (AdoHcyase) (EC 3.3.1.1) (S-adenosyl-L-homocysteine hydrolase) | 3 | 6.9 | 47.538 | 0 | 21.099 | 20.673 | 20.382 | 20.191 | 122.35 | 23 |
| P10818 | Cytochrome c oxidase subunit 6A1, mitochondrial (Cytochrome c oxidase polypeptide VIa-liver) | 5 | 63.1 | 12.301 | 0 | 22.962 | 20.512 | 20.851 | 21.213 | 63.576 | 48 |
| P10860 | Glutamate dehydrogenase 1, mitochondrial (GDH 1) (EC 1.4.1.3) (Memory-related gene 2 protein) (MRG-2) | 9 | 17 | 61.415 | 0 | 22.408 | 22.316 | 22.024 | 21.532 | 177.81 | 83 |
| P11517 | Hemoglobin subunit beta-2 (Beta-2-globin) (Hemoglobin beta chain, minor-form) (Hemoglobin beta-2 chain) | 18 | 64.6 | 15.982 | 0 | 23.820 | 22.539 | 22.480 | 23.933 | 323.31 | 158 |
| P11951 | Cytochrome c oxidase subunit 6C-2 (Cytochrome c oxidase polypeptide VIc-2) | 3 | 36.8 | 8.4548 | 0 | 21.380 | 20.638 | 21.995 | 21.032 | 38.138 | 31 |
| P11980 | Pyruvate kinase PKM (EC 2.7.1.40) (Pyruvate kinase muscle isozyme) | 15 | 40.1 | 57.817 | 0 | 21.201 | 21.607 | 22.322 | 21.651 | 323.31 | 84 |
| P12346 | Serotransferrin (Transferrin) (Beta-1 metal-binding globulin) (Liver regeneration-related protein LRRG03) (Siderophilin) | 17 | 28.4 | 76.394 | 0 | 23.432 | 22.072 | 22.598 | 23.061 | 317.85 | 139 |
| P16290 | Phosphoglycerate mutase 2 (EC 5.4.2.11) (EC 5.4.2.4) (BPG-dependent PGAM 2) (Muscle-specific phosphoglycerate mutase) (Phosphoglycerate mutase isozyme M) (PGAM-M) | 16 | 71.1 | 28.755 | 0 | 24.850 | 22.043 | 22.395 | 24.718 | 323.31 | 155 |
| P18163 | Long-chain-fatty-acid--CoA ligase 1 (EC 6.2.1.3) (Long-chain acyl-CoA synthetase 1) (LACS 1) (Long-chain-fatty-acid--CoA ligase, liver isozyme) | 19 | 37.1 | 78.178 | 0 | 22.625 | 20.985 | 22.051 | 23.068 | 323.31 | 109 |
| Q6AYX2 | L-lactate dehydrogenase (EC 1.1.1.27) | 22 | 72.3 | 35.712 | 0 | 25.218 | 21.718 | 24.290 | 25.267 | 323.31 | 195 |
| P19804 | Nucleoside diphosphate kinase B (NDK B) (NDP kinase B) (EC 2.7.4.6) (Histidine protein kinase NDKB) (EC 2.7.13.3) (P18) | 3 | 38.8 | 17.283 | 0 | 20.684 | 20.238 | 20.664 | 20.347 | 55.113 | 20 |
| P20760 | Ig gamma-2A chain C region | 10 | 32.6 | 35.185 | 0 | 21.385 | 21.895 | 21.710 | 22.167 | 172.46 | 82 |
| P32551 | Cytochrome b-c1 complex subunit 2, mitochondrial (Complex III subunit 2) (Core protein II) (Ubiquinol-cytochrome-c reductase complex core protein 2) | 4 | 20.1 | 48.396 | 0 | 20.881 | 20.751 | 21.586 | 20.145 | 323.31 | 29 |
| P46462 | Transitional endoplasmic reticulum ATPase (TER ATPase) (EC 3.6.4.6) (15S Mg(2+)-ATPase p97 subunit) (Valosin-containing protein) (VCP) | 23 | 41.8 | 89.348 | 0 | 23.210 | 21.345 | 22.001 | 22.780 | 323.31 | 136 |
| P55063 | Heat shock 70 kDa protein 1-like (Heat shock 70 kDa protein 1L) (Heat shock 70 kDa protein 3) (HSP70.3) | 27 | 43.7 | 70.548 | 0 | 23.699 | 21.752 | 22.712 | 23.508 | 323.31 | 202 |
| P63269 | Actin, gamma-enteric smooth muscle (Alpha-actin-3) (Gamma-2-actin) (Smooth muscle gamma-actin) [Cleaved into: Actin, gamma-enteric smooth muscle, intermediate form] | 20 | 60.6 | 41.876 | 0 | 21.984 | 22.717 | 21.715 | 20.746 | 173.1 | 38 |
| P68370 | Tubulin alpha-1A chain (Alpha-tubulin 1) (Tubulin alpha-1 chain) [Cleaved into: Detyrosinated tubulin alpha-1A chain] | 21 | 47 | 50.135 | 0 | 19.905 | 21.175 | 21.499 | 20.512 | 20.5 | 23 |
| Q4KLL5 | Ropporin-1 (Rhophilin-associated protein 1) | 10 | 58.5 | 23.96 | 0 | 23.465 | 21.397 | 21.980 | 23.057 | 322.26 | 67 |
| Q4KLZ6 | Triokinase/FMN cyclase (Bifunctional ATP-dependent dihydroxyacetone kinase/FAD-AMP lyase (cyclizing)) [Includes: ATP-dependent dihydroxyacetone kinase (DHA kinase) (EC 2.7.1.28) (EC 2.7.1.29) (Glycerone kinase) (Triokinase) (Triose kinase); FAD-AMP lyase (cyclizing) (EC 4.6.1.15) (FAD-AMP lyase (cyclic FMN forming)) (FMN cyclase)] | 8 | 27.7 | 59.443 | 0 | 21.716 | 20.453 | 21.012 | 21.072 | 202.32 | 59 |
| Q4QR77 | Protein FAM166A | 4 | 18.4 | 36.993 | 0 | 20.224 | 20.444 | 21.388 | 20.701 | 102.93 | 31 |
| Q4V8H5 | Aspartyl aminopeptidase (Aspartyl aminopeptidase, isoform CRA_c) | 9 | 24.2 | 52.555 | 0 | 22.990 | 21.077 | 21.502 | 22.698 | 323.31 | 60 |
| Q4V8P4 | Rsb-66 protein (Rsb-66 protein, isoform CRA_b) (Sperm acrosome-associated 9) | 3 | 26.2 | 19.465 | 0 | 22.096 | 21.405 | 21.291 | 21.317 | 323.31 | 56 |
| Q5RK28 | Normal mucosa of esophagus-specific gene 1 protein | 9 | 83.1 | 9.5981 | 0 | 23.175 | 21.312 | 23.009 | 24.095 | 275.93 | 78 |
| Q5XI62 | Protein MENT (Methylated in normal thymocytes protein) | 10 | 45.3 | 37.64 | 0 | 22.129 | 21.074 | 21.089 | 21.752 | 246.1 | 65 |
| Q5XIV1 | Phosphoglycerate kinase (EC 2.7.2.3) | 36 | 75.8 | 45.01 | 0 | 26.468 | 22.426 | 24.448 | 26.568 | 323.31 | 545 |
| Q68FR8 | Tubulin alpha-3 chain (Alpha-tubulin 3) [Cleaved into: Detyrosinated tubulin alpha-3 chain] | 24 | 54.7 | 49.959 | 0 | 26.260 | 22.054 | 25.308 | 26.152 | 323.31 | 363 |
| Q6AXN7 | 5'-nucleotidase, cytosolic IB | 21 | 39.5 | 64.922 | 0 | 22.062 | 20.507 | 22.363 | 22.780 | 323.31 | 134 |
| Q6AXR4 | Beta-hexosaminidase subunit beta (EC 3.2.1.52) (Beta-N-acetylhexosaminidase subunit beta) (Hexosaminidase subunit B) (N-acetyl-beta-glucosaminidase subunit beta) | 18 | 26.6 | 61.527 | 0 | 22.582 | 21.876 | 21.464 | 21.481 | 198.66 | 66 |
| Q6AXX6 | Redox-regulatory protein FAM213A (Peroxiredoxin-like 2 activated in M-CSF stimulated monocytes) (Protein PAMM) (Sperm head protein 1) | 14 | 50.7 | 25.763 | 0 | 22.103 | 22.055 | 23.310 | 23.618 | 240.02 | 79 |
| Q6AY07 | Fructose-bisphosphate aldolase (EC 4.1.2.13) | 20 | 56.6 | 39.491 | 0 | 23.972 | 22.122 | 23.037 | 24.506 | 323.31 | 220 |
| Q6AY30 | Saccharopine dehydrogenase-like oxidoreductase (EC 1.-.-.-) | 12 | 38.5 | 47.088 | 0 | 21.197 | 20.651 | 22.793 | 22.471 | 204.86 | 50 |
| Q6AYM2 | Tektin-2 (Tektin-t) (Testicular tektin) | 6 | 17 | 50.296 | 0 | 20.923 | 20.703 | 21.600 | 20.912 | 54.918 | 23 |
| Q6P502 | T-complex protein 1 subunit gamma (TCP-1-gamma) (CCT-gamma) | 11 | 30.1 | 60.646 | 0 | 21.359 | 20.191 | 21.238 | 20.783 | 186.2 | 52 |
| Q6P6V0 | Glucose-6-phosphate isomerase (GPI) (EC 5.3.1.9) (Autocrine motility factor) (AMF) (Neuroleukin) (NLK) (Phosphoglucose isomerase) (PGI) (Phosphohexose isomerase) (PHI) | 14 | 38.7 | 62.826 | 0 | 22.236 | 20.773 | 21.954 | 22.828 | 323.31 | 73 |
| Q6P762 | Alpha-mannosidase (EC 3.2.1.-) | 23 | 29.7 | 114.33 | 0 | 23.026 | 21.053 | 22.697 | 22.828 | 323.31 | 113 |
| Q6YFQ1 | Cytochrome c oxidase subunit 6B2 (Cytochrome c oxidase subunit VIb isoform 2) (COX VIb-2) (Cytochrome c oxidase subunit VIb, testis-specific isoform) | 4 | 46.6 | 10.472 | 0 | 21.130 | 20.959 | 20.676 | 21.936 | 28.904 | 28 |
| Q8VI04 | Isoaspartyl peptidase/L-asparaginase (EC 3.4.19.5) (EC 3.5.1.1) (Asparaginase-like protein 1) (Asparaginase-like sperm autoantigen) (Beta-aspartyl-peptidase) (Glial asparaginase) (Isoaspartyl dipeptidase) (L-asparagine amidohydrolase) [Cleaved into: Isoaspartyl peptidase/L-asparaginase alpha chain; Isoaspartyl peptidase/L-asparaginase beta chain] | 14 | 49.8 | 34.41 | 0 | 20.517 | 20.245 | 21.893 | 20.444 | 323.31 | 45 |
